# Religiosity, Spirituality, and Fatigue in Patients With Idiopathic Inflammatory Myopathies: A Brazilian Multicentric Case-Control Study

**DOI:** 10.7759/cureus.86858

**Published:** 2025-06-27

**Authors:** Jucier Gonçalves Júnior, Alexandre M Dos Santos, Romão A Sampaio, Thalita N Silva, Daniel Araujo, Estelita L Cândido, Samuel K Shinjo

**Affiliations:** 1 School of Medicine, Universidade Federal do Cariri, Barbalha, BRA; 2 School of Medicine, Universidade Regional do Cariri, Crato, BRA; 3 Division of Rheumatology, Faculdade de Medicina da Universidade de São Paulo, Universidade de São Paulo, São Paulo, BRA; 4 School of Medicine, Universidade Federal do Ceará, Fortaleza, BRA; 5 School of Medicine, Universidade Estadual do Ceará, Fortaleza, BRA; 6 Rheumatology, Immune, Pelotas, BRA; 7 Internal Medicine, Universidade Federal de Pelotas, Pelotas, BRA

**Keywords:** chronic fatigue, coping factors, dermatomyositis, health and spirituality, polymyositis, religiosity

## Abstract

Background

Despite advances in understanding and studying fatigue in idiopathic inflammatory myopathies (IIMs), no studies have addressed the literature that address the influence of religiosity and spirituality on fatigue in IIM patients.

Methodology

This multicenter, case-control study was conducted at four Brazilian institutions following the STROBE protocol. Data were collected between August 2022 and April 2023 using a semi-structured questionnaire that included sociodemographic information and the Duke University Religion Index (DUREL) with three domains (organizational religious affiliation (ORA), non-organizational religious affiliation (NORA), and intrinsic religiosity (IR)), Attitudes Related to Spirituality Scale (ARES), and Fatigue Severity Scale (FSS). Fatigue was defined as FSS scores ≥36. The sample was not probabilistic due to the rarity of IIMs. The control group was randomly selected from the same city where the case group was selected. Logistic regression was performed with the variables that showed statistical significance in the bivariate analysis.

Results

The IIM group was positively associated with IR (odds ratio (OR) = 2.70, 95% confidence interval (CI) = 0.20-1.77, p = 0.013), income of less than two minimum wages (OR = 12.21, 95% CI = 1.54-4.02, p < 0.001), between 2 and 4 (OR = 12.52, 95% CI = 1.31-3.73, p < 0.001), and being alone (OR = 0.18, 95% CI = -2.47 to -0.91, p < 0.001) in the multivariate analysis. The presence of fatigue (OR = 3.30, 95% CI = 0.56-1.82, p < 0.001) and comorbidities (OR = 3.36, 95% CI = 0.59-1.83, p < 0.001) were also associated with the case group in logistic regression. The IIM group with fatigue had higher religiosity (ORA, NORA, and IR) levels compared to patients without fatigue.

Conclusions

IIM with fatigue was associated with the presence of comorbidities, low socioeconomic levels, absence of a partner, and higher levels of religiosity.

## Introduction

Idiopathic inflammatory myopathies (IIMs) or systemic autoimmune myopathies are rare and severe autoimmune diseases caused by muscular, pulmonary, cardiac, and gastrointestinal impairments [1,2] that severely affect quality of life, with fatigue being a major limiting symptom [3].

Fatigue can be defined as difficulty in sustaining voluntary and prolonged motor tasks over an extended period of time [4,5] and is a common symptom in IIM patients in clinical practice [3]. Review studies have demonstrated a fatigue prevalence of up to 54%-65% in patients with IIMs [4]. Despite advances in the study of fatigue in rheumatic diseases, understanding the factors that cause fatigue, particularly in patients with IIMs, requires better theoretical contributions [3,4].

Spirituality can be understood as the search for meaning in life and its relationship with the sacred or the transcendent. Religiosity can be understood as adherence to practices that the individual believes in and follows, such as participation in a religious temple, reading religious books, and praying [6].

Literature demonstrates that religiosity and spirituality can be useful in coping with aspects such as pain, exhausting routines, promoting hope, and increasing therapeutic adherence in chronic diseases such as neoplasms [7], infectious diseases such as hepatitis [8], and mood disorders such as depression [9]. In diseases such as fibromyalgia, the impact of religiosity/spirituality on fatigue does not seem to be direct but rather encourages practices such as physical exercise and self-care, which lead to combating this condition [10,11].

There are currently only five studies that have investigated religiosity/spirituality in patients with rheumatic diseases. Of these, three studies involve non-autoimmune rheumatic diseases, that is, patients with fibromyalgia [10-12]; one study involves patients with chronic pain and osteoarthritis, frequent mechanical low back pain, and gout [13]; and only two studies involving DRAS, one with systemic autoimmune myopathies [14] and another with rheumatoid arthritis [15].

However, the impact of religiosity and spirituality on autoimmune rheumatic diseases, especially with regard to fatigue in IIMs, remains uncertain [3,4]. Thus, this study aims to determine whether there is a relationship between fatigue and spirituality/religiosity and encourage the creation of protocols to help manage fatigue in IIMs by addressing aspects of spirituality/religiosity. The primary aim of this study was to investigate whether there is an influence of spirituality/religiosity on fatigue in patients with IIMs. The secondary aim was to investigate whether there is an influence of other sociodemographic aspects on fatigue in patients with IIMs.

## Materials and methods

Study design

This was an observational, multicenter, case-control study, with a non-probabilistic sample conducted in four rheumatology outpatient clinics linked to Brazilian university hospitals. The choice of a non-probabilistic sample was due to the rarity of IIMs. The Strengthening the Reporting of Observational studies in Epidemiology (STROBE) protocol [12] for observational studies and the Checklist for Reporting Results of Internet E-Surveys (CHERRIES) protocol [13] for studies using online questionnaires were employed in this study.

Eligibility criteria

The present study included (i) patients who met the Bohan and Peter criteria [14] and the European League Against Rheumatism/American College of Rheumatology (EULAR/ACR) 2017 classification criteria [2] for IIMs (dermatomyositis, polymyositis, and immune-mediated necrotizing myopathy], or the criteria described by Connors et al. [1] for antisynthetase syndrome; (ii) patients who were ≥18 years old; and (iii) patients who agreed to participate in the study. All participants provided written informed consent. The control group comprises patients without autoimmune rheumatic diseases who were aged ≥18 years. The control group was collected from the same city where each case group was randomly recruited via an online questionnaire sent to groups of people on social media.

Study procedure

The data were collected by rheumatologists after routine medical consultations between August 2022 and April 2023. The instrument used consisted of an online e-survey, prepared with the data management tool REDCap^©^, version 11.2.5 (Vanderbilt University, USA). The tool was made available and maintained by a local institution in partnership with the REDCap-Brazil Consortium. The tool complied with all guidelines of the General Law for the Protection of Personal Data, Law No. 13.709/2018. The collected data were kept confidential.

The e-survey was divided into two stages. The first stage included demographic, socioeconomic, and educational data, including current age, sex, ethnicity, education, marital status (married, divorced, single, stable union, and widower), rent in minimum wages (≤2, 3-4, 5-10, and 11-20), education (illiterate, middle school incomplete, middle school complete, high school incomplete, high school complete, undergraduate degree, and bachelor’s degree), and comorbidities. The Brazilian minimum wage in effect at the time of collection was R$1,320.00 reais. The second stage consisted of three questionnaires in the following order: (a) Spirituality: Attitudes Related to Spirituality Scale (ARES) [6,15]; (b) Religiosity: Duke University Religion Index (DUREL) [16]; and (c) Perception of fatigue: Fatigue Severity Scale (FSS) [17].

ARES is an 11-item Likert scale with values ranging from 1 (totally disagree) to 5 (totally agree) for each item. The possible scores range from 11 to 55. The higher the score, the higher the spirituality index of the evaluated individuals [6,15].

DUREL comprises five items that capture the three dimensions of religiosity most closely related to health outcomes, namely, organizational religious affiliation (ORA), non-organizational religious affiliation (NORA), and intrinsic religiosity (IR). The first two items addressing ORA and NORA were extracted from large epidemiological studies conducted in the United States and were shown to be related to indicators of physical and mental health and social support. The other items refer to IR and are the three items in Hoge [18]. The IR scale was best related to the total score on this scale, social support, and health outcome. To analyze DUREL results, the scores in the three dimensions (ORA, NORA, and IR) need to be analyzed separately, and the scores of the three dimensions need not be added to the total score [19].

The DUREL and ARES scales were dichotomized to facilitate statistical analysis. On the DUREL scale, the items were scored from 1 to 6. “High” score was considered when the score was 4, 5, or 6. “Low” score was when the score was 1, 2, or 3 for evaluation of ORA (question 1) and NORA (question 2). In the IR assessment, questions 3-5 (composite score) were considered “high” (≥10) and “low” (<10).

The FSS consists of nine statements that rate the severity of a patient’s fatigue symptoms over the previous week. Patients scored each item on a scale of 1 to 7 based on the extent to which they agreed or disagreed with each statement (1 = strong disagreement, and 7 = strong agreement). The final score was obtained by summing the scores for each of the nine items. An FSS score ≥36 denoted fatigue [17]. The present questionnaire was selected based on its validity in the Brazilian population.

Data analysis

The data obtained from REDCap© were imported into the Epi Info software, version 7.2.5 (Centers for Disease Control and Prevention, USA). The values obtained from the fatigue (FSS), spirituality (ARES), and religiosity (DUREL) scales were evaluated using descriptive statistics, measures of variability, and central tendency.

In the second stage, we compared the fatigue averages of the IIM and control groups, stratified by sociodemographic factors and by the ARES and DUREL scales. The data were expressed as mean ± standard deviation, median (interquartile range = 25th-75th), or frequency (%). Because the three scales (FSS, ARES, and DUREL) did not have a normal distribution, Wilcoxon-Mann-Whitney and Kruskal-Wallis tests were used to compare the different median groups.

For the multivariate analysis, logistic regression test was performed, taking into account the variables that showed a statistically significant difference between cases and controls in the bivariate analysis (gender, age, income, marital status, fatigue, presence of comorbidities, and IR dichotomized), with the case group as the dependent variable.

When evaluating the contribution of each variable to the fatigue outcome, the chi-square test for dependence and odds ratio (OR) were used as a measure of association, taking into account a confidence interval (CI) of 95%. All hypothesis tests were performed at a significance level of 0.05. Cramer’s V calculated the effect size, and the phi coefficient calculated measures of association.

Ethical considerations

The ethical criteria outlined in the Declaration of Nuremberg, Declaration of Helsinki, Declaration of Belmont, the guidelines of the Brazilian National Health Council, and international standards were adhered to in the present study. All the procedures were approved by the Local Human Research Ethics Committee (approval number: CAAE 57910322.3.0000.0068).

## Results

Our study evaluated 168 patients with IIMs and 325 healthy controls. Fatigue levels in the IIM group were stratified by sociodemographic data. Those aged 31-59 years, caucasian, single, with income lower than two minimum wages or between 2-4 minimum wages, and those with comorbidities were associated with IIMs in bivariate analysis (Table 1).

**Table 1:** Characterization of fatigue in IIM patients and controls stratified by sociodemographic and comorbidity variables. IIM: idiopathic inflammatory myopathies; OR: odds ratio; CI: confidence interval

	IIM (N = 168)	Controls (N = 325)	OR (95% CI)	P-value	Phi coefficient	Cramer’s V
	Fatigue n (%)	Fatigue n (%)				
Sex						
Female	61 (47.3)	65 (32.9)	1.83 (1.16-2.89)	0.010	0.145	0.145
Male	20 (51.2)	37 (29.2)	-	0.008	0.198	0.198
Age (years)						
18–30	8 (47.1)	59 (33.6)	1.76 (0.64-4.80)	0.262	0.092	0.092
31–59	55 (51.4)	39 (30.9)	2.35 (1.38-4.02)	0.001	0.187	0.187
>60	18 (40.9)	4 (17.9)	3.23 (0.95-1.20)	0.051	0.237	0.237
Ethnicity						
Caucasian	42 (51.9)	59 (30.9)	2.40 (1.41-4.10)	0.001	0.198	0.198
Pardo	23 (45.1)	33 (31.5)	1.79 (0.90-3.56)	0.095	0.134	0.134
Black	15 (42.9)	5 (22.8)	2.55 (0.76-8.27)	0.121	0.205	0.205
Asian	1 (100.0)	5 (71.4)	-	0.537	0.218	0.218
Rent (minimum wage)						
≤2	38 (45.3)	15 (26.4)	2.31 (1.11-4.79)	0.022	0.192	0.192
3–4	30 (57.7)	16 (28.6)	3.40 (1.53-7.58)	0.002	0.294	0.294
5–10	11 (40.8)	39 (36.8)	1.18 (0.49-2.80)	0.705	0.033	0.033
11–20	2 (40.0)	32 (30.2)	1.54 (0.24-9.67)	0.641	0.044	0.044
Schooling						
Illiterate	1 (33.4)	0	-	<0.001	-	-
Middle school incomplete	10 (52.7)	1 (10.1)	-	0.008	0.363	0.363
Middle school complete	15 (57.7)	11 (61.1)	13.6 (1.51-122.8)	0.006	0.448	0.448
High school incomplete	1 (20.0)	0	5.80 (0.45-3,371.02)	0.103	0.349	0.349
High school complete	16 (38.9)	9 (38.0)	-	0.094	0.366	0.366
undergraduate degree	29 (63.7)	78 (34.6)	2.85 (0.73-11.06)	0.119	0.012	0.012
Bachelor’s degree	12 (36.4)	0	1.07 (0.50-2.30)	0.848	0.199	0.199
Marital status						
Married	38 (45.3)	22 (25.6)	2.40 (1.25-4.59)	0.007	0.206	0.206
Divorced	13 (50.0)	2 (20.0)	4.00 (0.70-22.55)	0.101	0.273	0.273
Single	21 (53.9)	69 (35.2)	2.14 (1.07-4.30)	0.028	0.143	0.143
Stable union	4 (57.2)	8 (25.9)	3.83 (0.70-20.97)	0.107	0.261	0.261
Widower	5 (41.7)	1 (50.0)	0.71 (0.03-14.34)	0.825	-0.059	-0.059
Comorbidities						
Yes	54 (51.5)	16 (27.2)	2.84 (1.42-5.67)	0.002	0.087	0.087
No	27 (42.9)	86 (32.4)	1.56 (0.89-2.75)	0.113	0.236	0.236

IR (OR = 2.70, 95% CI = 0.20-1.77, p = 0.013) and the presence of fatigue (OR = 3.30, 95% CI = 0.56-1.82, p < 0.001) were associated with the IIM group. Low income (less than two minimum wages: OR = 12.21, 95% CI = 1.54-4.02, p < 0.001) or between 2-4 (OR = 12.52, 95% CI = 1.31-3.73, p < 0.001), being single (OR = 0.18, 95% CI = -2.47 to -0.91, p < 0.001), and the presence of comorbidities (OR = 3.36, 95% CI = 0.59-1.83, p < 0.001) were also associated with the case group (Table 2).

**Table 2:** Multivariate analysis. IR: intrinsic religiosity; OR: odds ratio; CI: confidence interval

	OR	95% CI	P-value
IR	2.70	0.20-1.77	0.013
Rent (<2)	16.21	1.54-4.02	<0.001
Rent (3–4)	12.52	1.31-3.73	<0.001
Single	0.18	-2.47--0.91	<0.001
Fatigue	3.36	0.56-1.82	<0.001
Comorbidities	3.30	0.59-1.83	<0.001

There was a 7.5 times chance among individuals with fatigue of presenting with high levels of ORA (OR = 7.59, 95% CI = 3.78-15.76, p < 0.001) and almost half of those without fatigue (OR = 3.28, 95% CI = 1.91-5.70, p < 0.001). In the analysis of the NORA scale, a 9.3 times chance (OR = 37, 95% CI = 3.98-24.86, p < 0.001) was observed for the group with fatigue and 6.6 times for the group without fatigue (OR = 6.68, 95% CI = 3.30-14.72, p < 0.001). Patients in the IIM and control groups with fatigue presented with high levels of IR, with a chance of 1.33 and 1.12 times, respectively, without statistical significance (Table 3).

**Table 3:** Characterization of religiosity by DUREL in IIM patients and controls stratified by fatigue. DUREL: Duke University Religion Index; FFS: Fatigue Severity Scale; IIM: idiopathic inflammatory myopathies; IR: intrinsic religiosity; NORA: non-organizational religious affiliation; ORA: organizational religious affiliation; OR: odds ratio; CI: confidence interval

IIM (N = 168)				Controls (N = 325)				
	Low religiosity, n (%)	High religiosity, n (%)	Low religiosity, n (%)	High religiosity, n (%)	OR (95% CI)	P-value	Phi coefficient	Cramer’s V
ORA								
Fatigue								
Yes	25 (30.8%)	56 (69.2%)	79 (77.4%)	23 (22.6%)	7.59 (3.78-15.76)	<0.001	0.062	0.062
No	36 (41.3%)	51 (58.7%)	156 (69.9%)	67 (30.1%)	3.28 (1.91-5.70)	<0.001	0.272	0.272
NORA								
Fatigue								
Yes	8 (9.8%)	73 (90.2%)	52 (50.9%)	50 (49.9%)	9.37 (3.98-24.86)	<0.001	0.065	0.065
No	11 (41.3%)	76 (58.7%)	110 (69.9%)	113 (30.1%)	6.68 (3.30-14.72)	<0.001	0.187	0.187
IR								
Fatigue								
Yes	5 (41.7%)	76 (48.8%)	36 (29.8%)	66 (32.6%)	1.33 (0.40-4.37)	0.637	0.296	0.296
No	7 (58.3%)	80 (51.2%)	85 (70.2%)	138 (67.4%)	1.12 (0.69-1.83)	0.625	0.347	0.347

## Discussion

To our knowledge, this is the first study to evaluate the association between religiosity/spirituality and fatigue in IIM patients. Fatigue was more prevalent in the IIM group, who were single, of white ethnicity, of low socioeconomic status, and had comorbidities. The advantages of this study include its multicenter design, 1:3 matching between cases and controls, and the large sample size, considering that it is a rare disease.

The IIM group was more associated with the presence of fatigue, IR, being single, comorbidities, and low socioeconomic status. Patients with fatigue had higher religiosity levels on the DUREL (ORA, NORA, and IR) scale when compared with those without fatigue. Spirituality was comparable between groups.

Fatigue has been studied as a multifactorial component of rheumatic diseases, depending on its biological, social, and behavioral aspects [20]. A recent review noted that factors, such as the presence of comorbidities, may contribute to worsening fatigue in rheumatic diseases such as rheumatoid arthritis, spondyloarthritis, fibromyalgia, and osteoarthritis; however, there is no consensus in the literature [21].

A Brazilian cohort that evaluated 57 patients with rheumatoid arthritis demonstrated higher levels of fatigue in low-income individuals [22]. In Egypt, fatigue was more prevalent between the fourth and fifth decades of life [23]. A Canadian study showed an important impact of fatigue on the work among patients with rheumatic diseases, notably on absenteeism (percentage of time lost due to health reasons) and presenteeism (percentage of incapacity at work due to health reasons) [24]. In this context, the literature also highlights that the absence of social support and work overload may be possible causes of fatigue in rheumatic diseases [20].

However, we did not find any studies that reported the influence of sociodemographic factors on fatigue in IIM patients. In an extensive literature review on fatigue in rheumatic diseases, Beckers et al. [25] did not find any studies on IIMs. Thus, we hypothesized that young, single individuals with low socioeconomic status and IIM may present a favorable situation for fatigue due to the association between these factors. Furthermore, it is important to emphasize that epidemiological surveys in the Brazilian population have shown a prevalence of 83% to 88% of the population as self-declared religious practitioners [25,26].

The literature has demonstrated religiosity as a tool for coping with symptoms that affect the quality of life of patients with chronic diseases, such as cancer [7], pain syndromes [27], infections such as hepatitis [8], chronic kidney disease, and diabetes mellitus [28]. One of the most accepted theories is that individuals use religiosity/spirituality as a way to improve self-esteem, maintain hope [29] in treatment, and increase adherence to non-pharmacological measures and pharmacological treatment [30]. An Israeli study demonstrated higher levels of religiosity in individuals with fibromyalgia who practiced more physical exercise and, in turn, had lower fatigue scores [10]. A German study [11] among 102 patients with fibromyalgia demonstrated that the presence of religious beliefs was negatively associated with the choice of “ignoring the disease” as a coping strategy. Thus, we hypothesized that higher rates of IR in patients with IIM and fatigue may be a coping strategy to deal with these and other symptoms of the disease. Other rheumatic diseases, such as rheumatoid arthritis, also demonstrate a positive influence of religiosity on biological and health aspects [31]. However, cultural differences deserve to be highlighted. A study of a German and Polish population with amyotrophic lateral sclerosis found no difference in religiosity/spirituality in aspects inherent to quality of life [32].

This study has a few limitations, including (i) the non-homogeneity of the sample due to the socioeconomic and cultural differences of the Brazilian population and its large territory. (ii) Non-validation of the DUREL scale for non-Eastern religions. (iii) The impossibility of formulating a cause-and-effect relationship due to the limitations of the study type. Finally, (iv) the lack of control for confounding factors such as mood disturbance and disease activity/severity in the case group.

## Conclusions

Comparing the IIM group with the controls showed that single patients with low socioeconomic status, presence of comorbidities, fatigue, and IR are associated with the case group. The IIM group with fatigue had higher religiosity levels by ORA, NORA, and IR scale when compared with those without fatigue. Other longitudinal studies are needed to evaluate the impact of spirituality/religiosity on fatigue across different religions in IIM patients. In addition, the use of validated instruments for the cross-cultural assessment of spirituality/religiosity in the Brazilian population should be promoted quantitatively.

## References

[1] Connors GR, Christopher-Stine L, Oddis CV, Danoff SK (2010). Interstitial lung disease associated with the idiopathic inflammatory myopathies: what progress has been made in the past 35 years?. Chest.

[2] Lundberg IE, Fujimoto M, Vencovsky J (2021). Idiopathic inflammatory myopathies. Nat Rev Dis Primers.

[3] de Oliveira DS, de Souza JM, Shinjo SK (2019). Beyond medicine: physical exercise should be always considered in patients with systemic autoimmune myopathies. Autoimmun Rev.

[4] Misse RG, Borges IB, Dos Santos AM, Gupta L, Shinjo SK (2021). Effect of exercise training on fatigue and pain in patients with systemic autoimmune myopathies: a systematic review. Autoimmun Rev.

[5] Ricci G, Fontanelli L, Torri F, Schirinzi E, Siciliano G (2022). Fatigue as a common signature of inflammatory myopathies: clinical aspects and care. Clin Exp Rheumatol.

[6] Forti S, Serbena CA, Scaduto AA (2020). [Spirituality/religiousity measurement and health in Brazil: a systematic review]. Cien Saude Colet.

[7] Arbinaga F, Mendoza-Sierra MI, Bohórquez MR, Verjano-Cuellar MI, Torres-Rosado L, Romero-Pérez N (2021). Spirituality, religiosity and coping strategies among Spanish people diagnosed with cancer. J Relig Health.

[8] Sohail MM, Mahmood QK, Sher F, Saud M, Mas'udah S, Ida R (2020). Coping through religiosity, spirituality and social support among Muslim chronic hepatitis patients. J Relig Health.

[9] Miller L, Wickramaratne P, Gameroff MJ, Sage M, Tenke CE, Weissman MM (2012). Religiosity and major depression in adults at high risk: a ten-year prospective study. Am J Psychiatry.

[10] Aloush V, Gurevich-Shapiro A, Hazan E, Furer V, Elkayam O, Ablin JN (2021). Relationship between religiosity, spirituality and physical and mental outcomes in fibromyalgia patients. Clin Exp Rheumatol.

[11] Braun A, Evdokimov D, Frank J, Pauli P, Wabel T, Üçeyler N, Sommer C (2022). Relevance of religiosity for coping strategies and disability in patients with fibromyalgia syndrome. J Relig Health.

[12] Biccheri E, Roussiau N, Mambet-Doué C (2016). Fibromyalgia, spirituality, coping and quality of life. J Relig Health.

[13] Gudz I, Pais-Ribeiro J, Ferreira-Valente A (2021). [Association between religiosity, resilience and subjective well-being in people with chronic pain]. Psic Saúde Doenças.

[14] Gonçalves Júnior J, Dos Santos AM, Sampaio RA (2024). Spirituality, religiosity, and mental health in patients with idiopathic inflammatory myopathies: a Brazilian multicentric case-control study. Int J Environ Res Public Health.

[15] Bartlett SJ, Piedmont R, Bilderback A, Matsumoto AK, Bathon JM (2003). Spirituality, well-being, and quality of life in people with rheumatoid arthritis. Arthritis Rheum.

[16] Cuschieri S (2019). The STROBE guidelines. Saudi J Anaesth.

[17] Eysenbach G (2004). Improving the quality of Web surveys: the Checklist for Reporting Results of Internet E-Surveys (CHERRIES). J Med Internet Res.

[18] Bohan A, Peter JB (1975). Polymyositis and dermatomyositis (first of two parts). N Engl J Med.

[19] Braghetta CC, Gorenstein C, Wang YP (2021). Development of an instrument to assess spirituality: reliability and validation of the Attitudes Related to Spirituality Scale (ARES). Front Psychol.

[20] Taunay TCD, Gondim FAA, Macêdo DS (2012). [Validity of the Brazilian version of the Duke Religious Index (DUREL)]. Arch Clin Psychiatry (São Paulo).

[21] Hewlett S, Dures E, Almeida C (2011). Measures of fatigue: Bristol Rheumatoid Arthritis Fatigue Multi-Dimensional Questionnaire (BRAF MDQ), Bristol Rheumatoid Arthritis Fatigue Numerical Rating Scales (BRAF NRS) for severity, effect, and coping, Chalder Fatigue Questionnaire (CFQ), Checklist Individual Strength (CIS20R and CIS8R), Fatigue Severity Scale (FSS), Functional Assessment Chronic Illness Therapy (Fatigue) (FACIT-F), Multi-Dimensional Assessment of Fatigue (MAF), Multi-Dimensional Fatigue Inventory (MFI), Pediatric Quality Of Life (PedsQL) Multi-Dimensional Fatigue Scale, Profile of Fatigue (ProF), Short Form 36 Vitality Subscale (SF-36 VT), and Visual Analog Scales (VAS). Arthritis Care Res (Hoboken).

[22] Koenig H, Parkerson GR Jr, Meador KG (1997). Religion index for psychiatric research. Am J Psychiatry.

[23] Moreira-Almeida A, Peres MF, Aloe F (2008). [Portuguese version of Duke Religious Index: DUREL]. Arch Clin Psychiatry (São Paulo).

[24] Hewlett S, Chalder T, Choy E (2011). Fatigue in rheumatoid arthritis: time for a conceptual model. Rheumatology (Oxford).

[25] Beckers E, Hermans K, Van Tubergen A, Boonen A (2023). Fatigue in patients with rheumatic and musculoskeletal diseases: a scoping review on definitions, measurement instruments, determinants, consequences and interventions. RMD Open.

[26] Diniz LR, Balsamo S, Souza TY, Muniz LF, Martins WR, Mota LM (2017). Measuring fatigue with multiple instruments in a Brazilian cohort of early rheumatoid arthritis patients. Rev Bras Reumatol Engl Ed.

[27] Mortada M, Abdul-Sattar A, Gossec L (2015). Fatigue in Egyptian patients with rheumatic diseases: a qualitative study. Health Qual Life Outcomes.

[28] Enns MW, Bernstein CN, Kroeker K (2018). The association of fatigue, pain, depression and anxiety with work and activity impairment in immune mediated inflammatory diseases. PLoS One.

[29] Majda A, Szul N, Kołodziej K, Wojcieszek A, Pucko Z, Bakun K (2022). Influence of spirituality and religiosity of cancer patients on their quality of life. Int J Environ Res Public Health.

[30] Onyishi CN, Eseadi C, Ilechukwu LC, Okoro KN, Okolie CN, Egbule E, Asogwa E (2022). Potential influences of religiosity and religious coping strategies on people with diabetes. World J Clin Cases.

[31] Gonçalves Júnior J, Siqueira LC, de Alencar Junior AE, Shinjo SK (2025). Spirituality and religiosity in rheumatic diseases: a systematic review [in press]. J Relig Health.

[32] Ciećwierska K, Lulé D, Helczyk O (2023). Religiosity in patients with amyotrophic lateral sclerosis, a cross-country comparison. Qual Life Res.

